# Adverse drug reactions, adherence, and virologic outcomes in adult patients on dolutegravir-based antiretroviral therapy at a tertiary hospital, southeast Nigeria

**DOI:** 10.4314/gmj.v58i1.14

**Published:** 2024-03

**Authors:** Lawrence U Ogbonnaya, Cosmas K Onah, Benedict N Azuogu, Christian O Akpa, Kingsley C Okeke, Violet N Nwachukwu, Adaeze Stephen-Emeya, Irene U Asaga, Chukwuma D Umeokonkwo

**Affiliations:** 1 Department of Community Medicine, College of Health Sciences Ebonyi State University Abakaliki Nigeria; 2 Department of Community Medicine, Alex Ekwueme Federal University Teaching Hospital Abakaliki, Ebonyi State Nigeria

**Keywords:** Human Immunodeficiency Virus, Antiretroviral Therapy, Dolutegravir

## Abstract

**Objective:**

To assess the adherence, adverse drug reactions (ADR), and virologic outcomes of dolutegravir-based antiretroviral therapy.

**Design:**

This was a retrospective chart review.

**Setting:**

A tertiary health facility-based study in Abakaliki, Nigeria.

**Participants:**

Five hundred and fifteen (515) adult patients on dolutegravir were selected using a Random Number Generator. Demographic and clinical data were extracted from patients' case notes and analysed with IBM-SPSS version-25.

**Main outcome measures:**

Adherence to dolutegravir, ADRs, virologic outcome, and change in Body Mass Index (BMI) were estimated.

**Results:**

The mean age of the patients was 45.5±10.8 years; 68.2% of them were females; 97.1% of them had good self-reported adherence. The majority (82.9%) of them reported no ADRs and among those (17.1%) that did, headache (9.7%), body-itching (3.1%), and skin rash (2.7%) dominated. Most achieved viral suppression (94.4%) and did not have detectable viral particles (57.4%). There was a significant increase in the BMI of the patients with a mean weight increase of 0.9kg, a mean BMI increase of 0.3 kg/m^2^, and a 2.6% increase in the prevalence of overweight and obesity.

**Conclusions:**

Patients on dolutegravir reported low ADRs, good self-reported adherence, and a high viral suppression rate. However, dolutegravir is associated with weight gain. We recommend widespread use and more population-wide studies to elucidate the dolutegravir-associated weight gain.

**Funding:**

None declared

## Introduction

Human immunodeficiency virus (HIV) is an infection that attacks and weakens the body's immunity against opportunistic infections such as tuberculosis, fungal and bacterial infections, and some cancers.^1^ HIV is a major global public health issue, with an estimated 38.4 million people living with the disease and 650,000 HIV-related deaths in 2021.^2^,^3^ The infection is a chronic condition with no cure, which can be managed on a long-term basis with preventive measures, diagnosis, treatment, and care, including opportunistic infections, enabling HIV-positive people to lead long and healthy lives.^4^–^6^ Treatment of HIV with antiretroviral drugs (ARVs) is known to lower viral load (VL), fight infections by other pathogens, improve the quality of life, and lower the chances of transmitting HIV to other people.^7^

The treatment has been identified as a critical step to ending the Acquired Immune Deficiency Syndrome (AIDS) epidemic and making HIV transmission rare.^8^

Driven by the efforts to improve drug efficacy and toxicity profiles and ensure patients' adherence to treatment, retention in care, and reduction in the emergence of antimicrobial resistance, ARVs' discovery and development have evolved recently.^9^,^10^ This has given rise to several classes of drugs based on different modes of action, including nucleoside/nucleotide reverse transcriptase inhibitors (NRTIs), non-nucleoside reverse transcriptase inhibitors (NNRTIs), protease inhibitors (PI), and integrase strand transfer inhibitors (INSTIs).^11^ Currently, there are more than 30 ARVs.^9^

The principle of HIV drug treatment includes the use of regimens of a cocktail of at least three ARVs from two classes, referred to as antiretroviral therapy (ART).^12^ The aim of this combination is to launch multiple channels of attack on the virus, quickly reduce the VL, improve patient response and guard against antimicrobial resistance.^12^ Since 2016, the World Health Organization (WHO) has recommended a “treat all strategy”, meaning that all HIV-positive people should be provided with ARVs, irrespective of WHO clinical stage and at any CD4 cell count.^13^,^14^ Furthermore, ART initiation should be rapidly offered to HIV-positive people on the same day following a confirmed HIV diagnosis and clinical assessment for those who are ready to start.^13^

In 2018, WHO recommended dolutegravir (DTG), an INSTI, in combination with two NRTI and boosted PI, in combination with an optimized two NRTI backbone, as the preferred first-line and second-line regimens, respectively, for HIV-positive people.^13^ Due to evolving evidence supporting the efficacy, safety and tolerability of DTG, the WHO recommended the use of DTG as the preferred first-line and second-line treatment for all populations, including pregnant women and those of childbearing potential in 2021.^13^,^15^ DTG-based regimens are efficacious in treatment-naïve and treatment-experienced patients infected with HIV due to a more rapid viral suppression and higher genetic resistance barrier, when compared with NNRTIs.^16^,^17^

Successful ART requires that all medications be taken as prescribed to prevent drug resistance and treatment failure.^7^ The treatment goal is to reduce the VL to undetectable levels of less than 50 copies/ml. The persistence of detectable viral particles greater than 1000 copies/ml in HIV-positive people on ART is an indicator of inadequate treatment and virological failure.^1^ Virological failure refers to VL >1000 copies/mL after two consecutive VL measurements 3 months apart, with adherence support following the first viral load test (VLT).^13^ ART switch is recommended after the first VL >1000 copies/mL or after the second VL >50 to <1000 copies/mL for those receiving NNRTI-based ART. Viral load testing provides an early and accurate indication of treatment failure and the need to switch from first-line to second-line drugs, reducing the accumulation of drug-resistant mutations and improving clinical outcomes.

Nigeria ranks third among countries with the highest burden of HIV infection globally. According to the 2019 National HIV, AIDS Indicator, and Impact Survey (NAIIS), the national HIV prevalence was 1.5%, and 1.9 million people were living with HIV and AIDS.^18^ Nigeria has, since 2018, adopted the WHO recommendation on the use of DTG-based ART as the first line for the treatment of HIV-positive people. According to WHO recommendations, there is a need in countries transitioning to DTG to monitor the safety and virologic outcome of ART and the emergence of resistance to DTG-based ART.^13^ Further research is required to better understand DTG-associated weight gain and the pattern of fat deposition. The aim of this study, therefore, was to describe the adverse drug reaction (ADR), virologic outcome and adherence among patients on DTG-based ART. It is hoped that findings from this study will inform the improved quality of ART services to HIV-positive people.

## Methods

### Study setting

This study was conducted at the Communicable Disease Control and Research Centre (CDCRC), Alex Ekwueme Federal University Teaching Hospital Abakaliki (AEFUTHA), Nigeria. The CDCRC is a clinic for the treatment of adult male and non-pregnant female patients infected with HIV and other sexually transmitted diseases. The clinic uses the WHO-recommended “test and treat strategy”. The transitioning of patients to a DTG-based regimen at the clinic started in 2018 but was majorly done in 2019. The criteria for this transition included being on the first-line regimen, being virally suppressed and being treatment naïve. At the time of this study, all patients who met these conditions had been switched to a DTG, and DTG-based ART was the only first-line ART regimen used in the clinic.

The schedule of patient appointments at the clinic is: at 2 weeks after enrolment and initiation on DTG, at 4 weeks following the first appointment, and at every 12 weeks thereafter. This schedule is adapted to monitor the safety of the drug and adherence after the initiation of ART. The indications for VLT at the CDCRC are in line with the recommendations of the WHO and the national guidelines for HIV prevention, treatment and care,^13^,^19^ and include:

- Baseline test (at six months after ART initiation),
- Routine test (every 12 months thereafter),
- Clinical failure and immunologic failure confirmation (3 – 6 months after intense adherence counselling), and iv. A repeat test in cases of indeterminate results for evidence-informed decision-making.

### Study population and design

The study was conducted among adult patients accessing ART services using retrospective chart review. The study population was all patients who were on DTG at the time of the study.

### Sample size, inclusion, and exclusion criteria

Using the Cochran formula (n= Z_α_^2^pq/d^2^) for sample size,^20^ a standard normal deviate, Z_α_, of 1.96, a prevalence of VL suppression (VLS), p, of 80.9% reported in NAIIS 2018,^18^ and a precision, d, of 3.5%, a sample size, n, of 485, was estimated. However, a total of 515 patients were reviewed for a better representation of the study population and more accurate results. Patients who were on DTG and have had at least one VLT result done at least 6 months after commencing DTG were included. Patients who defaulted or skipped appointments for more than three months at any point in time after the commencement of DTG or after being switched to DTG-based ART for any other documented reasons except for observed ADRs reported to be due to intake of DTG-based ART were excluded. This was done to avoid the dilution effect of such defaulting on ADR reports, self-reported adherence to DTG and virologic outcome.

### Sampling Technique

Using their enrolment numbers, the number of patients accessing ART at CDCRC was obtained from the medical records unit of CDCRC AEFUTHA. This number was fed into an online Random Number Generator^21^ as the maximum number; 1 was fed in as the minimum. The application was instructed to generate numbers in ascending order and, without allowing repeats, that are equivalent to the desired sample size. With the generated numbers, the folders of the patients were retrieved from the medical records and screened for inclusion in the study. Any patients not meeting the inclusion criteria were replaced using the same method until the sample size was made up.

### Data instrument and collection

The data were collected through a review of each patient's hospital folder and HIV care card and extraction of documented information using a pro forma. The data extracted from the folders were the patients' biodata, including their age, sex, height, weight at the commencement or transition to DTG, and weight at the time of study. Others include ART regimen at initiation, total duration of intake of ART, duration of intake of DTG-based ART, number of clinic attendance since the commencement of DTG or transition to it, reported ADRs, reported adherence to DTG-based ART and documented VLT results from baseline (for patients who were transitioned to DTG) or first follow up VLT (for patients who were initiated on DTG). The review was conducted in the month of May 2022.

### Data Analysis

Data were entered into the IBM-SPSS version 25 and cleaned. Data were transformed by recoding and computing them into relevant variables for analysis. Data were analysed using IBM-SPSS Statistics version 25, and charts were drawn using Microsoft Excel Spreadsheet version 2019. Chi-square, and paired samples t-test were done at the confidence level of 95% and p-value of 0.05.

### Measurement of outcome variables

#### Adverse drug reaction

Adverse drug reaction due to DTG-based ART was assessed as per known and unknown ADRs related to intake of DTG. This was determined as the proportion of patients on a DTG-based regimen who ever reported any known and/or unknown ADR due to DTG.

#### Virologic outcome

The virologic outcome was assessed as the proportion of patients who were virally suppressed (VL <1000 copies/mL) following initiation on or switch to a DTG-based regimen; patients with VL above >1,000 copies/ml were categorised as virally unsuppressed.

#### Adherence

Adherence was assessed as “good” or “fair” based on the proportion of patients with reported “good” or “fair” adherence to ART since initiation on or switch to a DTG-based regimen. Reported good adherence is defined as taking up to 29 (≥95%) doses of once-daily DTG-based ART over thirty days, while reported fair and poor adherence amounts to taking less than 29 (<95%) doses over the same period.

### Ethical approval and consent to participate

This study was approved by the Health Research and Ethics Committee of Alex Ekwueme Federal University Teaching Hospital Abakaliki, Nigeria, with approval number AEFUTHA/REC/VOL.3/2022/019. Secondary level data was collected from treatment folders and cards; the authors did not have any contact with the patients.

## Results

At the time of initiation of DTG, the majority (73.2%) of the patients were ART-experienced and were on ART comprising two NRTIs and one NNRTI, while 26.8% were treatment-naïve (Table 1). The mean age of the patients was 45.5±10.8 years. Over two-thirds (68.2%) of them were females (Table 1).

**Table 1:** Sociodemographic characteristics and ART intake profile of adult patients on dolutegravir-based regimen at CDCRC AEFUTHA

Variable	Frequency N=515 n (%)
**Age in years**	
**< 40**	140 (27.2)
**40 - 49**	207 (40.2)
**50 - 59**	116 (22.5)
**≥ 60**	52 (10.1)
**Mean age**	45.5±10.8*^ms^*
**Sex**	
**Female**	351 (68.2)
**Male**	164 (31.8)
**ART history at start of DTG**	
**ART-experienced**	377 (73.2)
**ART-naive**	138 (26.8)
**Duration of intake of any ART (in years)** ^***mi***^	
**Patients transitioned to DTG (N = 377)**	10.1 (3.4)*^mi^*
**Patients initiated on DTG (N = 138)**	1.5 (1.5)*^mi^*
**Overall (N = 515)**	9.0 (8.7)*^mi^*
**Duration of intake of DTG-based ART (in years)**	
**Patients transitioned to DTG (N = 377)**	2.8 (0.4)*^mi^*
**Patients initiated on DTG (N = 138)**	1.5 (1.5)*^mi^*
**Overall (N = 515)**	2.7 (0.8)*^mi^*
**Number of clinic attendance since the commencement of DTG**	
**< 11**	131 (25.4)
**11 - 15**	323 (62.7)
**> 15**	61 (11.8)
**Mean attendance**	12.2±3.1*^ms^*

ms = mean ± standard deviation; mi = median (interquartile range)

Overall, the median duration of any ART was 9 years with an interquartile range (IQR) of 8.7, [9 (8.7)] years. Disaggregated into two, the median duration of ART by treatment-experienced patients was 10.1 (3.4) years, while that of the treatment-naïve patients initiated on DTG was 1.5 (1.5) years. The overall median duration of intake of DTG was 2.7 (0.8) years. Those who were transitioned to DTG-based ART have taken it for 2.8 (0.4) years, while those who were initiated on it have taken it for 1.5 (1.4) years. Majority (62.7%) of the patients have attended between 11 and 15 ART clinics since the commencement of DTG (Table 1).

A majority (82.9%) of the patients did not report any ADRs since they started taking DTG. Of those (88/515; 17.1%) with any ADR reports, headache (50/515; 9.7%), body itching (16/515; 3.1%), skin rash (14/515; 2.7%), abdominal pain (9/515; 1.7%), and fatigue (8/515; 1.6%) were the major complaints (Table 2). Self-reported good adherence to DTG-based ART was observed in 97.1% of the patients.

**Table 2:** Self-reported adverse drug reaction and adherence to DTG-based ART among adult patients at CDCRC AEFUTHA

Variable	Frequency N=515 n (%)
**Ever reported any ADR to DTG-based ART**	
**No**	427 (82.9)
**Yes**	88 (17.1)
**Type of reported ADR** *	
**Headache**	50 (9.7)
**Itching**	16 (3.1)
**Skin rash**	14 (2.7)
**Abdominal pain**	9 (1.7)
**Fatigue**	8 (1.6)
**Anorexia**	7 (1.4)
**Insomnia**	5 (1.0)
**Diarrhoea**	4 (0.8)
**Dizziness**	2 (0.4)
**Nausea**	1 (0.2)
**Vomiting**	1 (0.2)
**Nightmare**	1 (0.2)
**Self-reported adherence to DTG-based ART**	**N=515**
**Good**	500 (97.1)
**Fair to poor**	15 (2.9)

* Multiple responses were allowed

Table 3 shows comparisons of the mean weight and Body Mass Index (BMI) of the patients at the time of the study with their values at the start of DTG. The mean weight of the patients at the time of the study (65.0±15.4Kg) was significantly higher than their weight at the start of DTG (64.1±15.2Kg; *t*=2.804; 95% Confidence Interval [CI]: -1.53 – -0.27; *p*=0.005). Similarly, the BMI of the patients at the time of the study (24.8±5.6 Kg/M^2^) was significantly higher than their BMI at the start of DTG (24.5±5.5 Kg/M^2^; *t*=-2.865; 95% CI: 0.60–0.11; *p*=0.004).

**Table 3:** Effect of dolutegravir-based ART on weight and Body Mass Index patients at CDCRC AEFUTHA

Variable	N	Period of measurement		t-test*^a^*	95% CI	p-value
		**Start of DTG**	**After use of DTG**			
**Mean weight (Kg)**	515	64.1±15.2	65.0±15.4	-2.804	-1.53 – -0.27	0.005*
**Mean BMI (Kg/M^2^)**	515	24.5±5.5	24.8±5.6	-2.865	-0.60 – -0.11	0.004*

Kg = Kilogram; M = Metre; BMI = Body Mass Index = DTG, Dolutegravir; CI = Confidence Interval; *^a^* = Paired Samples T-test

* Statistical significance

**Figure 1:**
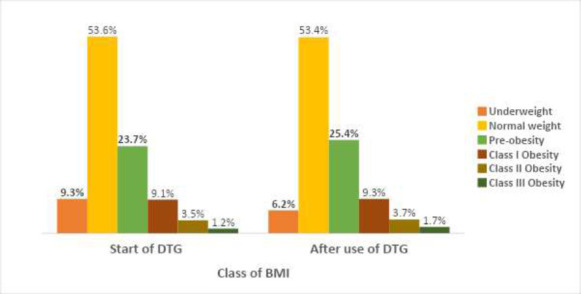
BMI status of patients on dolutegravir-based ART at CDCRC AEFUTHA at start of DTG and currently

Figure 1 shows that the proportion of patients who were underweight at the start of DTG (9.3%) decreased by 3.1% to 6.2%, while that of patients with normal weight (53.2%) increased by 0.4% to 53.6%. The prevalence of overweight/pre-obesity (23.7%) and obesity (13.8%) increased respectively by 1.7% and 0.9%) to 25.4% and 14.7%, compared to the start of DTG. A Wilcoxon signed-rank test showed a statistically significant change in the BMI of the patients (Z = -3.101, p = 0.002)

Table 4 shows the status of VL of patients at the start of DTG and at the first, second and third follow-up VLT, grouped according to the laboratory report and the degree of viral suppression. All (515) of the patients had done the first VLT, while only 399 and 46 of them had done the second and third VLT, respectively, following the commencement of DTG. At the start of DTG, 95.8% of the ART-experienced patients were virally suppressed. At the first, second and third follow-up VLT, 95.7%, 96.2% and 91.3% of the patients, respectively, were virally suppressed, giving a combined prevalence of 94.4%. Amongst the patients in the three test periods, 47.8%, 63.4%, and 60.9%, respectively, had undetectable viral particles (TND) in their plasma, giving a combined prevalence rate of 57.4%. Compared to the start of DTG, the proportion of patients with undetectable viral particles was 23.2% higher.

**Table 4:** Status of viral load of adult patients on DTG-based ART at CDCRC AEFUTHA according to laboratory result categories

Period of VLT	N	Viral Load Status					

		Suppressed (<1000 copies/mL) n (%)					Not suppressed (>1000 copies/mL) n (%)
		TND	<20	20-39	40-149	150-1000	
**Start of DTG (ART-experienced patients only)**	377	129 (34.2)	104 (27.6)	65 (17.2)	47 (12.5)	16 (4.2)	16 (4.2)
**First follow-up VLT (All patients)**	515	246 (47.8)	48 (9.3)	118 (22.9)	65 (12.6)	16 (3.1)	22 (4.3)
**Second follow-up VLT (All patients)**	339	215 (63.4)	10 (2.9)	61 (18.0)	31 (9.1)	9 (2.7)	13 (3.8)
**Third follow-up VLT (All patients)**	46	28 (60.9)	2 (4.3)	9 (19.6)	1 (2.2)	2 (4.3)	4 (8.7)

VLT = Viral Load Test; TND = Target Not Detected, is a state of undetectable viral particles in patient's plasma

## Discussion

This study has demonstrated self-reported good treatment adherence in 97.1%, absence of ADR in 82.9%, and viral suppression in 94.4% of patients on DTG-based ART. The high level of self-reported treatment adherence is a crucial factor in the effectiveness of HIV treatment, as consistent adherence helps suppress the virus and prevent the development of drug resistance. The absence of ADR in the majority of patients indicates that DTG is well-tolerated by a significant proportion of them.

These findings corroborate those of previous studies establishing good adherence, high safety, and viral suppression in patients on DTG-based ART.^16^,^17^,^22^,^23^ Further, the findings are in support of previous reports that DTG is very well tolerated, safe, has infrequent drug-drug interactions and tends to be protective against treatment discontinuation due to ADRs.^16^,^23^ These qualities are the reason DTG is now a preferred component of first-line ART in all populations infected with HIV.^24^,^25^

Only 17.1% of our patients reported at least an ADR while taking DTG-based ART. This prevalence of ADR may not necessarily be attributed solely to DTG, considering that several factors can contribute to the occurrence of ADRs in patients on HIV treatment. This is a positive aspect of the medication, as it implies that patients can take DTG with minimal discomfort or side effects. It further means that patients are less likely to stop taking their medication because of side effects, improving the chances of long-term treatment success. The prevalence of ADR in this study is approximately half of the 33% reported previously in Uganda.^26^ However, this is much higher than the 2.24% reported in Brazil.^27^ The discrepancies in these reports may be due to variations in pharmacogenetic characteristics of the patients studied, which can affect the pharmacokinetics of the drug in them,^28^ and the reporting biases that the patients may have had. It could be due to other factors such as additional medications, drug interactions, comorbidities, and psychosocial factors. Despite several reports of the high safety and tolerability of DTG, the relatively high prevalence of ADR in some settings underscores a need for continued pharmacovigilance of DTG in all populations to better understand the properties of the drug for its continued widespread use.

Headache, itching, and skin rash dominated the symptoms reported in this study. Previously, neuropsychiatric side effects such as insomnia, headache and dizziness, and hypersensitivity skin reactions like itchy rash have been documented as common ADRs associated with the use of DTG.^16^,^23^,^29^,^30^ These findings mean that DTG-based regimens occasionally fall short of the quest for ARVs that are safe, efficacious and not prone to microbial resistance, which has remained the driving force behind the search for newer drugs.^9^,^10^ These symptoms, however, are most often mild and self-limiting,^16^,^23^ and so are expected to have minimal negative influence on tolerability of the drug, adherence and continuation of their use. According to Patel et al, DTG has lower odds of discontinuation due to ADRs compared to treatments with other ARVs.^31^ As efforts to develop newer drugs for HIV treatment continue, emphasis should be laid on these observations to produce safer, more efficacious drugs with good tolerability.

We recorded a viral suppression of 94.4% and a prevalence of undetectable viral particles of 57.4%, a value that is 23.2% higher than that of the ART-experienced patients at the commencement of DTG. These findings lend credence to the assertion of high viral suppression of DTG.^32^,^33^ Being much higher than the 80.9% viral suppression reported in 2018 Nigeria NAIIS,^18^ our finding suggests an improvement in HIV control efforts.

However, this finding is local and may not reflect the national statistics. The 94.4% viral suppression in our study is comparable with the 2025 UNAID target of 95%,^34^, and this is commendable. It means that the level of HIV in the patient's blood is so low that it is undetectable or maintained at a level that does not harm the immune system. Viral suppression is a critical goal in HIV treatment because it helps control the virus's replication, reducing the risk of transmission and improving overall health. While the current viral suppression rate is close to the UNAIDS target, efforts should indeed continue to reach and even exceed this goal. The key lies in a combination of a comprehensive implementation of HIV treatment guidelines, patient education, regular monitoring, and addressing barriers to adherence.

This study showed a significant increase in BMI, and a 2.6% increase in the prevalence of overweight and obesity compared to the start of the DTG-based ART. A significant increase in BMI suggests that patients on DTG-based ART experienced weight gain throughout their treatment. It's important to note that this increase in BMI may be explained by the expected gain in body weight of sick persons returning to normal health. In the case of HIV patients, improved health due to viral suppression, changes in appetite or eating habits, and changes in lifestyle could be responsible. However, body weight gain with the use of DTG has previously been reported.^35^ According to Thivalapill et al, transition to DTG is associated with an increase in BMI.^36^ In Greece, Pantazis et al reported a more pronounced BMI increase of 12.4% in patients on INSTI regimens, compared to 8.5% and 6.5% increases in patients on PIs and NNRTI respectively.^37^

Body weight gain with the use of new ARVs, especially INSTIs, is considered a metabolic event due to multifactorial causes, such as race, sex, advanced disease, CD4 cell count, HIV viral load, and previous weight loss.^25^ Previous studies have shown that weight gain in patients on DTG is significantly higher than in those on non-DTG-based ART.^38^,^39^ Consequently, more population-wide surveys of patients on DTG are needed to elucidate the observed increase in weight. Beyond research to generate knew knowledge, healthcare providers need to closely monitor changes in weight and BMI in patients on ART, as weight gain can have both positive and negative consequences. While weight gain might be beneficial for patients who were underweight or experiencing wasting syndrome before treatment, it could pose a health risk for those who become overweight or obese. Weight monitoring by healthcare providers will help to appropriately manage the weight gain while at the same time treating the HIV disease.

## Conclusion

This study demonstrated that DTG is highly safe and effective and is associated with good treatment adherence. These findings reinforce the wide adoption of DTG as the preferred component of ART in all populations infected with HIV. This study further showed a significant increase in BMI when compared with BMI at the start of the therapy. Although this increase may be partly explained by the expected gain in weight of sick persons returning to normal health, the authors are not unaware that unmeasured factors such as diet, co-morbidities and co-medications might have influenced this observation. There is a need, therefore, for more population-wide studies that would take these variables into cognizance to better elucidate the role of DTG in weight gain.
